# Noninvasive prediction of EGFR 19Del and 21L858R subtypes in lung adenocarcinoma: a comparative study of logistic regression and decision tree models

**DOI:** 10.3389/fonc.2025.1642253

**Published:** 2025-09-18

**Authors:** Peng Han, Dai Zhang, Wenjun Yao, MengYu Lv, YunHong Qian, Hong Zhao

**Affiliations:** ^1^ Department of Radiology, The Second Affiliated Hospital of Anhui Medical University, Hefei, China; ^2^ Medical Imaging Research Center, Anhui Medical University, Hefei, China

**Keywords:** EGFR, lung adenocarcinoma, decision tree model, logistic regression model, NRI

## Abstract

**Objective:**

Despite the increasing interest in radiogenomic prediction, few studies have directly compared the performance of logistic regression and decision tree models in distinguishing epidermal growth factor receptor (EGFR) mutation subtypes. This study provides the first systematic comparison of the predictive performance of these two models in identifying exon 19 deletions (19Del) and exon 21 L858R point mutations (21L858R) in patients with lung adenocarcinoma. By leveraging imaging and clinical parameters, we aimed to address a critical gap in the literature by establishing an optimal prediction model and providing a noninvasive tool to support personalized treatment strategies for patients with unknown EGFR mutation status.

**Materials and methods:**

We retrospectively collected clinical and radiological data from 193 patients with histologically confirmed lung adenocarcinoma who were admitted to the Second Affiliated Hospital of Anhui Medical University between May 2018 and June 2024. Based on EGFR genotyping results, patients were stratified into two groups: the EGFR 19Del mutation group and the EGFR 21L858R mutation group. Comparative statistical analyses—including Student’s *t*-test, Mann–Whitney *U* test, chi-square test, or Fisher’s exact test—were performed to evaluate differences in clinical and CT imaging characteristics between groups. Variables with P < 0.05 in the univariate analysis were subsequently included in both logistic regression and decision tree models to identify independent predictors of EGFR mutation subtype. Model performance was assessed using ROC curve analysis. The area under the curve (AUC) was calculated for each model, and their predictive accuracy was further compared using DeLong’s test, net reclassification improvement (NRI), and integrated discrimination improvement (IDI).

**Results:**

In the decision tree model, age and brain metastasis emerged as key decision nodes for differentiating 19Del and 21L858R mutations, with an AUC of 0.712 (95% CI: 0.639–0.785). In contrast, the logistic regression model identified age, pleural thickening, lymphadenopathy, and brain metastasis as independent predictors, achieving a higher AUC of 0.740 (95% CI: 0.671–0.810). The NRI and IDI values were 0.498 (*P* < 0.001, 95% CI: 0.238–0.758) and 0.043 (*P* = 0.004, 95% CI: 0.013–0.072), respectively, suggesting improved reclassification and discrimination by the logistic model. However, DeLong’s test revealed no statistically significant difference between the AUCs of the two models (Z = 1.314, *P* = 0.189).

**Conclusion:**

Both logistic regression and decision tree models demonstrated value in predicting EGFR 19Del and 21L858R mutations in lung adenocarcinoma, each offering distinct methodological advantages. The logistic regression model exhibited higher interpretability and statistical robustness, making it well-suited for clinical decision-making. Meanwhile, the decision tree model offered superior visual clarity and intuitive structure, which may enhance practical utility. A combined modeling approach that harnesses the strengths of both methods may provide a more accurate and comprehensive tool for early mutation identification and individualized treatment planning in patients with lung adenocarcinoma.

## 1 Introduction

Lung adenocarcinoma (LUAD), a predominant histological subtype of non-small cell lung cancer (NSCLC), continues to pose a substantial global health burden, with 2024 estimates (Global Cancer Observatory preliminary data) indicating approximately 1.1 million new cases and 720,000 deaths annually worldwide, accounting for 45-50% of total NSCLC mortality (1, 2). Among its molecular drivers, mutations in the epidermal growth factor receptor (EGFR) gene are the most frequently observed, particularly in Asian populations, where their prevalence underscores the critical need for genotype-guided treatment strategies. The two most common EGFR mutation subtypes—exon 19 deletion (19Del) and exon 21 L858R point mutation (21L858R)—collectively account for approximately 85% of all EGFR mutations, and exhibit distinct therapeutic sensitivities (3–5).

Accumulating evidence suggests that patients harboring EGFR 19Del mutations generally derive greater clinical benefit from EGFR tyrosine kinase inhibitors (TKIs) compared to conventional chemotherapy (6, 7), while the response to TKIs in those with L858R mutations appears more variable, with some studies indicating comparable or even superior outcomes with cytotoxic chemotherapy (8, 9). These findings highlight the necessity of accurately identifying EGFR mutation subtypes to enable more precise and individualized therapeutic decisions (10, 11).

Currently, tissue biopsy remains the gold standard for determining EGFR mutation status. However, this invasive procedure is associated with potential complications such as hemorrhage, infection, and pneumothorax. Moreover, due to intratumoral heterogeneity, small biopsy samples may not fully reflect the genomic landscape of the entire tumor (12). Consequently, there is a pressing need for noninvasive, rapid, and reliable methods to predict EGFR mutation subtypes in clinical settings.

With the rapid evolution of medical imaging technologies, radiological assessment has emerged as an essential tool not only for diagnosis and treatment monitoring but also for exploring molecular correlates through imaging biomarkers (13–15). Although numerous studies have investigated the potential of imaging features for predicting EGFR mutation status in LUAD (16, 17), the comparative efficacy of logistic regression versus decision tree models in discriminating EGFR mutation subtypes (particularly 19Del vs. 21L858R) remains underexplored, representing a critical knowledge gap this study seeks to address. Therefore, in this study, we sought to develop and compare logistic regression and decision tree models based on clinical parameters and CT imaging features to noninvasively predict EGFR mutation subtype status in patients with lung adenocarcinoma.

## 2 Materials and methods

### 2.1 General information

This retrospective study was approved by the Institutional Review Board of the Second Affiliated Hospital of Anhui Medical University (Approval No. YX2023-212), with the requirement for written informed consent waived. A total of 1,200 patients with pathologically confirmed lung adenocarcinoma and documented epidermal growth factor receptor (EGFR) mutations were initially screened. These patients were diagnosed and treated at the Second Affiliated Hospital of Anhui Medical University between November 2018 and June 2024.

The inclusion criteria were as follows:

1. histopathological diagnosis of primary lung adenocarcinoma;
2. presence of EGFR mutations limited to exon 19 deletions (19Del) or exon 21 L858R point mutations (21L858R) as determined by gene testing;
3. availability of preoperative chest CT scan data;
4. no history of other malignancies;
5. no history of preoperative radiotherapy or chemotherapy.

**Figure 1:**
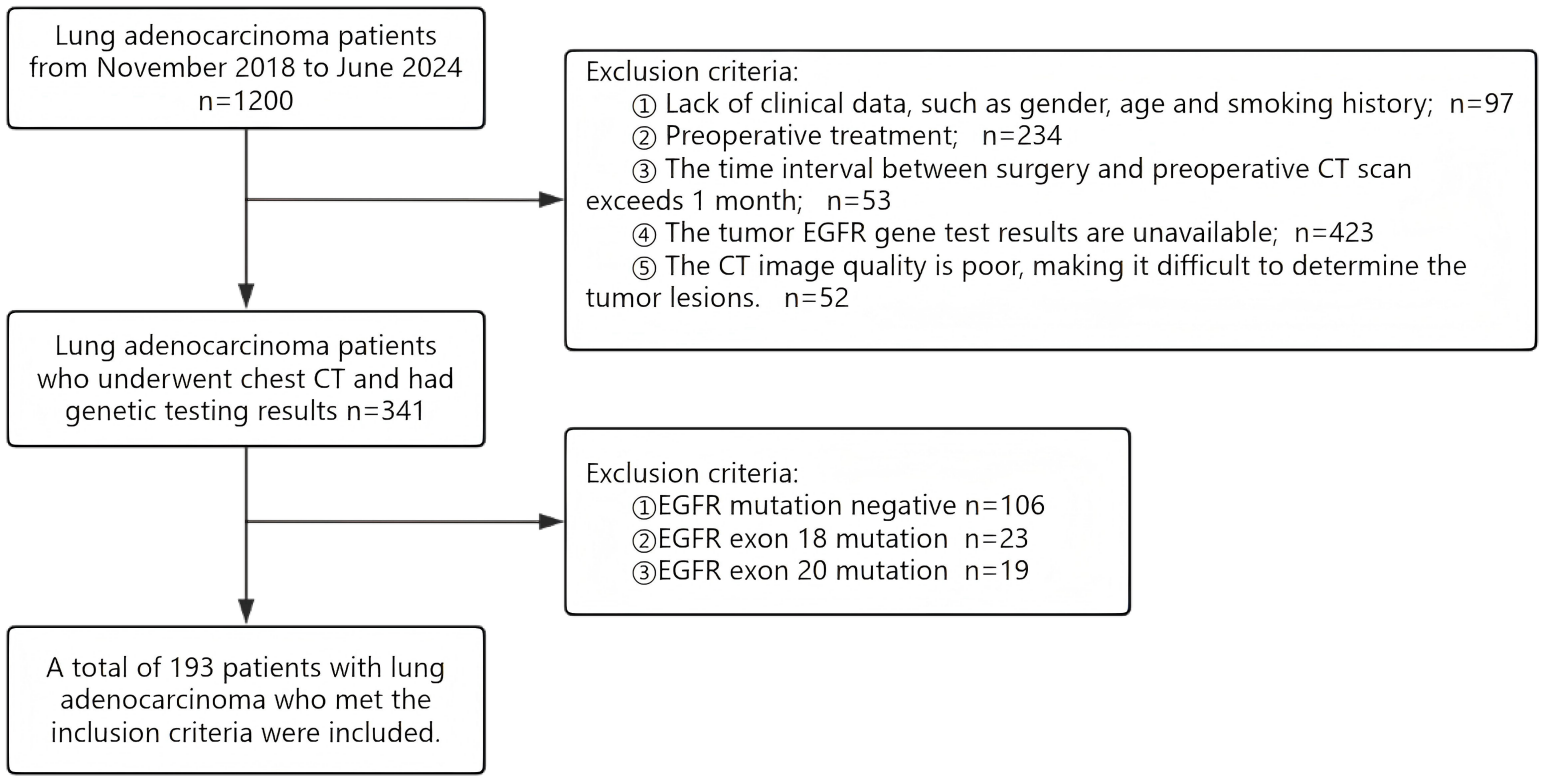
Flow chart of case enrollment.

Exclusion criteria included:

1. incomplete clinical information (e.g., gender, age, smoking history);
2. receipt of any treatment prior to surgery;
3. time interval exceeding one month between the preoperative CT scan and surgical resection;
4. missing EGFR mutation subtype results;
5. The CT image quality is poor, making it difficult to determine the tumor lesions. Ultimately, 193 patients with lung adenocarcinoma EGFR mutation subtypes were included in this study. The screening process is shown in the figure (**Figure 1**).

### 2.2 CT scan parameters

All patients underwent chest computed tomography (CT) scanning using a SOMATOM Force 128-slice scanner (Siemens Healthineers, Erlangen, Germany). The imaging protocol was standardized as follows: tube voltage was set at 120 kV, with a tube current ranging from 150 to 200 mA. The display field of view (DFOV) was 350 mm. Axial images were acquired with a slice thickness and interslice gap of 5 mm. For image reconstruction, a slice thickness of 1.25 mm and a reconstruction interval of 1.25 mm were applied, with a matrix size of 512 × 512. These parameters ensured optimal spatial resolution for the assessment of pulmonary and mediastinal structures.

### 2.3 CT image interpretation

Following communication and approval from the hospital’s administrative and medical records departments, clinical case data were retrieved through the institution’s electronic medical record system. Computed tomography (CT) images were independently reviewed by two board-certified radiologists, each with five years of diagnostic experience. During the image interpretation process, both radiologists were blinded to the patients’ clinical data and mutation status to minimize bias. Interobserver agreement for qualitative CT features was assessed using Cohen’s Kappa statistic, with detailed results provided in Supplementary Material **Supplementary Table S1**. In instances of initial disagreement, a consensus was reached through discussion. The following clinical and radiological features were systematically documented. Clinical variables included sex, age, and smoking history. CT images were evaluated using standard lung window settings (window width: 1500 HU; window level: −700 HU) and mediastinal window settings (window width: 350 HU; window level: 40 HU). The assessed CT features encompassed tumor morphology (including lesion type, maximum diameter, lobulation, and spiculation), internal characteristics (such as calcification, necrosis, cavitation, and air bronchogram), pleural abnormalities (including thickening, retraction, and effusion), peritumoral changes (e.g., emphysema and vascular convergence), lymph node involvement, distant metastases (brain, liver, bone, and contralateral lung), and CT attenuation values.

### 2.4 Detection of EGFR mutations in tumor specimens

EGFR mutation analysis was conducted in the Department of Pathology at the Second Affiliated Hospital of Anhui Medical University. Mutations in exons 19 and 21 of the EGFR gene were detected using the PCR-ARMS (amplification refractory mutation system) technique with a commercially available human EGFR mutation detection kit (Beijing SinoMD Gene Detection Technology Co., Ltd., Beijing, China).

Cases harboring exon 19 deletions were classified as the EGFR 19Del mutation group, while those exhibiting the exon 21 L858R point mutation were assigned to the EGFR 21L858R mutation group.

### 2.5 Statistical analysis methods

Statistical analyses were performed using SPSS software version 27.0 (IBM Corp., Armonk, NY, USA), with a two-tailed P-value < 0.05 considered indicative of statistical significance. For continuous variables, the independent samples t-test was applied to data with a normal distribution, while the Mann–Whitney U test was used for non-normally distributed data. Categorical variables were analyzed using the chi-square test or Fisher’s exact test, as appropriate. Variables demonstrating statistical significance (P < 0.05) in univariate analyses were subsequently entered into a multivariate logistic regression model to identify independent predictors associated with 19Del and 21L858R mutations in patients with lung adenocarcinoma. A classification-based decision tree algorithm was employed to construct the decision tree model, with node splitting based on chi-square values. Internal validation was conducted via 10-fold cross-validation to assess model robustness. Receiver operating characteristic (ROC) curves were generated for both the logistic regression and decision tree models, and the areas under the curve (AUCs) were calculated to evaluate predictive performance. Comparative analyses between the two models were further conducted using the DeLong test, net reclassification improvement (NRI), and integrated discrimination improvement (IDI) metrics.

## 3 Results

### 3.1 Clinical and imaging characteristics of patients

Patients with the EGFR 21L858R mutation exhibited a significantly higher prevalence of pleural thickening and brain metastasis compared to those with the EGFR 19Del mutation (P < 0.05). Conversely, lymphadenopathy was more frequently observed in the EGFR 19Del mutation group (P < 0.05). Additionally, age was found to be negatively associated with the presence of EGFR 19Del mutation. However, no statistically significant differences were identified between the two groups in terms of general clinical characteristics (e.g., sex, smoking history) or CT imaging features, including tumor type, location, maximum diameter, lobulation, and other morphological signs (P > 0.05), as detailed in **Table 1**.

**Table 1:** Association between clinical and CT imaging features and EGFR 19Del or 21L858R mutations.

Variable	EGFR mutation subtype		Statistic value	*P* value
	19Del n(%)	21L858R n(%)		
Gender			0.01	0.921
Female	49(55.06)	58(55.77)		
Male	40(44.94)	46(44.23)		
Age(year)	58.000(51.0,67.0)	69.000(58.0,73.0)	4.437	<0.001*
Smoking history			2.853	0.091
Absent	79(88.76)	83(79.81)		
Present	10(11.24)	21(20.19)		
Tumor location			0.306	0.580
Central	16(17.98)	22(21.15)		
Peripheral	73(82.02)	82(78.85)		
Tumor size	34.200(23.8,48.4)	35.000(25.9,53.3)	0.352	0.725
Lobular sign			0.432	0.511
Absent	10(11.24)	15(14.42)		
Present	79(88.76)	89(85.58)		
Spike Sign			0.016	0.900
Absent	36(40.45)	43(41.35)		
Present	53(59.55)	61(58.65)		
Edge			0.170	0.680
Clarity	48(53.93)	53(50.96)		
Unclear	41(46.07)	51(49.04)		
Air bronchogram			1.325	0.250
Absent	51(57.30)	68(65.38)		
Present	38(42.70)	36(34.62)		
Bubble sign			0.039	0.843
Absent	67(75.28)	77(74.04)		
Present	22(24.72)	27(25.96)		
Peripheralemphysema			1.182	0.277
Absent	80(89.89)	88(84.62)		
Present	9(10.11)	16(15.38)		
Vascular clustering			0.534	0.465
Absent	12(13.48)	18(17.31)		
Present	77(86.52)	86(82.69)		
Necrosis in tumor			0.232	0.630
Absent	51(57.30)	56(53.85)		
Present	38(42.70)	48(46.15)		
Pleural traction			0.029	0.865
Absent	23(25.84)	28(26.92)		
Present	66(74.16)	76(73.08)		
Pleural thickening			10.418	<0.001*
Absent	64(71.91)	51(49.04)		
Present	25(28.09)	53(50.94)		
Calcificationintumor			2.407	0.121
Absent	77(86.52)	81(77.88)		
Present	12(13.48)	23(22.12)		
Pleural effusion			0.642	0.423
Absent	63(70.79)	68(65.38)		
Present	26(29.21)	36(34.62)		
Inanition			0.341	0.559
Absent	81(91.01)	97(93.27)		
Present	8(8.99)	7(6.73)		
Swollen lymph nodes			4.628	0.031*
Absent	30(33.71)	51(49.04)		
Present	59(66.29)	53(50.96)		
CT value	38.57 ± 10.76	39.72 ± 9.71	0.787	0.432
Bone metastasis			1.513	0.219
Absent	55(61.80)	73(70.19)		
Present	34(38.20)	31(29.81)		
Brain metastases			6.129	0.013*
Absent	61(68.54)	53(50.96)		
Present	28(31.46)	51(49.04)		
Liver metastasis			2.006	0.157
Absent	80(89.89)	99(95.19)		
Present	9(10.11)	5(4.81)		
Lung metastasis			0.264	0.607
Absent	83(93.26)	94(91.26)		
Present	6(6.74)	9(8.74)		

*P < 0.05.

### 3.2 Establishment of multi-factor logistic regression model

**Figure 2:**
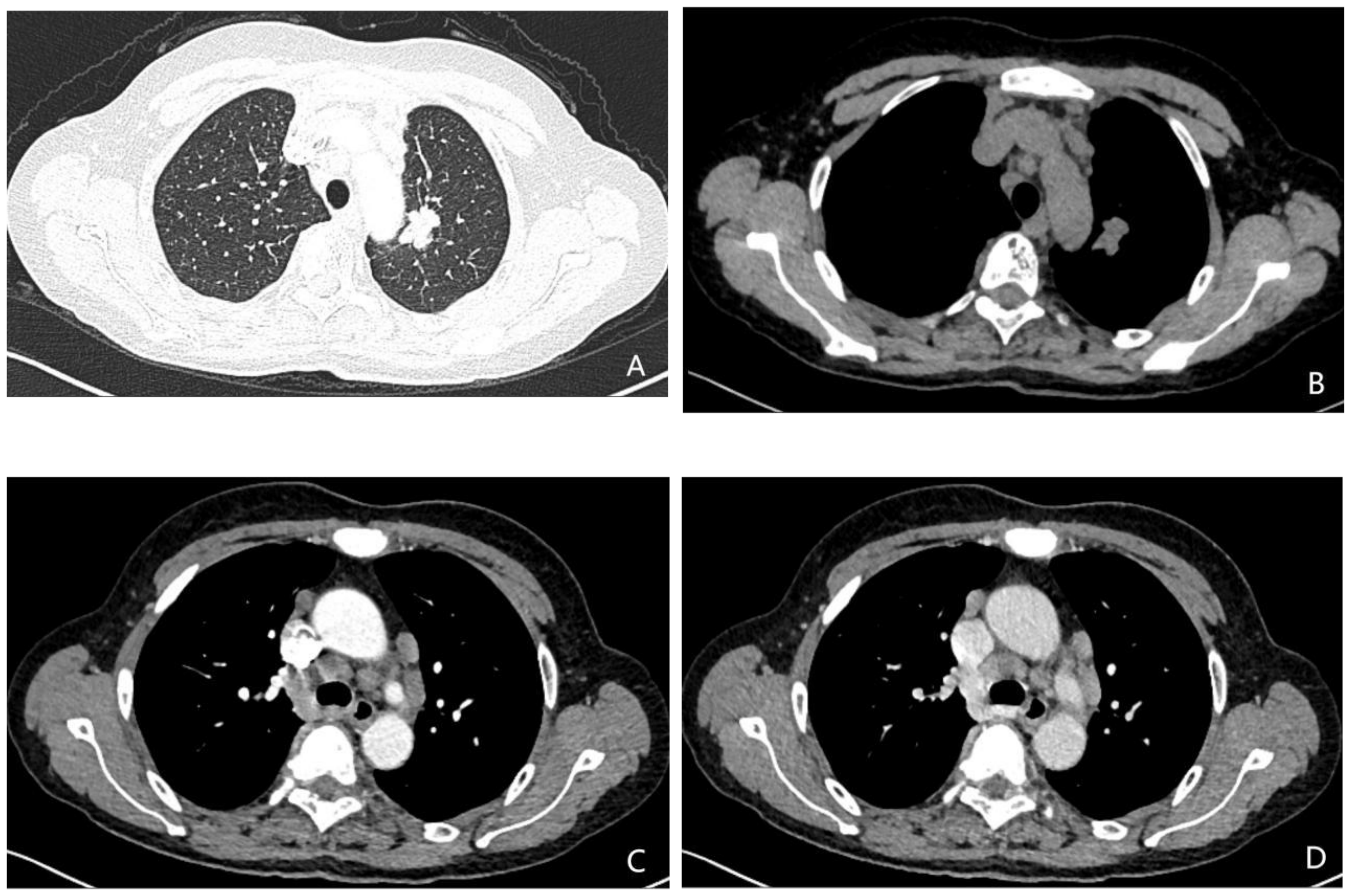
**(A-D)** A 58-year-old female patient with lung adenocarcinoma, the genetic test result was EGFR 19Del mutation (+). **(A-B)** Unenhanced CT demonstrates an irregular soft-tissue density shadow in the left upper lobe, measuring 25.3 mm in maximum diameter with lobulated margins. **(C, D)** Contrast-enhanced CT shows multiple enlarged lymph nodes adjacent to the trachea and aorta during arterial and venous phases.

**Figure 3:**
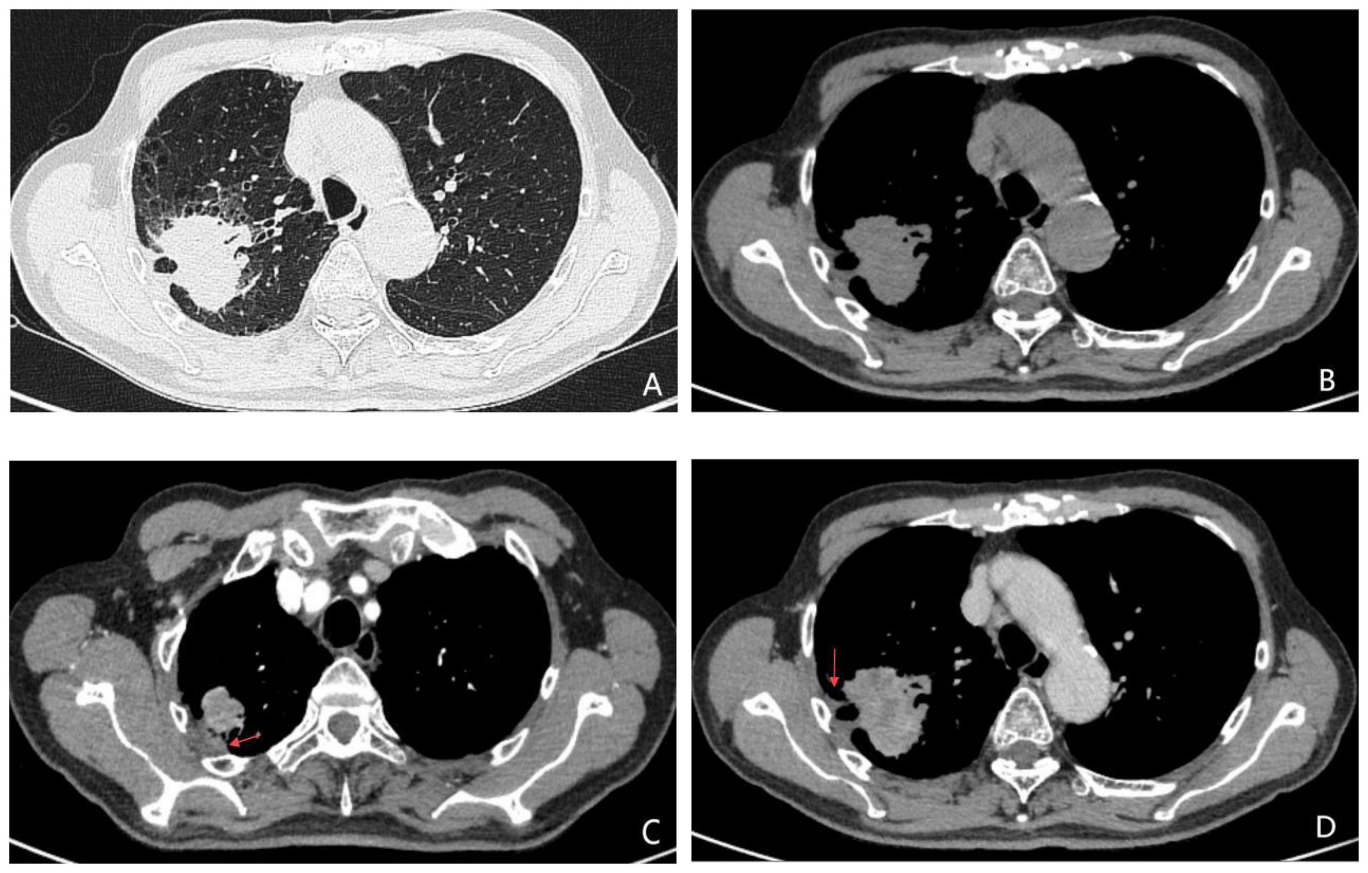
**(A-D)** A 74-year-old male patient with lung adenocarcinoma, the genetic testing result was EGFR 21L858R mutation (+). Unenhanced CT images **(A, B)** demonstrate an irregular soft-tissue nodule with indistinct margins and adjacent pleural thickening in the right upper lobe. Contrast-enhanced CT during arterial **(C)** and venous **(D)** phases reveals heterogeneous marked enhancement of the lesion, with no significant lymphadenopathy in the mediastinum.

Variables identified as statistically significant in the univariate analysis were subsequently included in the multivariate logistic regression model. The results revealed that age (OR = 1.065, *P* = 0.001; 95% CI: 1.031–1.101), brain metastasis (OR = 1.975, *P* = 0.036; 95% CI: 1.045–3.730), and pleural thickening (OR = 2.124,*P* = 0.026; 95% CI: 1.096–4.117) were positively associated with an increased likelihood of harboring the EGFR 21L858R mutation. In contrast, lymphadenopathy was more frequently associated with the EGFR 19Del mutation (OR = 0.462, *P* = 0.019; 95% CI: 0.242–0.881). These findings are summarized in **Table 2**, with representative clinical imaging examples presented in **Figures 2** and **3**.

**Table 2:** Multivariate logistic regression model associated with EGFR19DeL and 21L858R mutations.

Variable	B	s.e	Wald	Sig.	Exp(B)		95% CI
Age	0.063	0.017	14.068	0.001	1.065	1.031	1.101
Pleural thickening	0.753	0.338	4.978	0.026	2.124	1.096	4.117
Lymphadenopathy	-0.772	0.329	5.497	0.019	0.462	0.242	0.881
Brain metastases	0.680	0.324	4.396	0.036	1.975	1.045	3.73

### 3.3 Building a decision tree model

**Figure 4:**
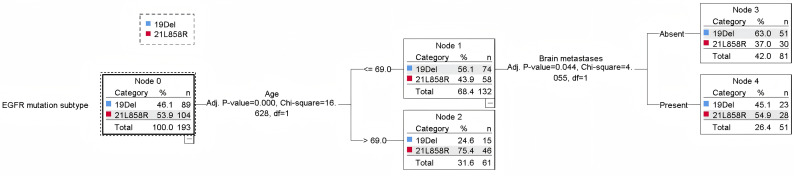
Decision tree model.

The results of the decision tree model, constructed using the classification decision tree algorithm, are illustrated in **Figure 4**. This model comprises three levels and five nodes, three of which are terminal nodes. Age and brain metastasis emerged as the key factors in predicting EGFR 19Del and 21L858R mutation status in patients with lung adenocarcinoma. At the first level, the model was stratified by age, highlighting a strong association between age and EGFR mutation status. Specifically,

among patients aged ≤69 years, 56.1% exhibited the EGFR 19Del mutation and 43.9% the EGFR 21L858R mutation; among those aged >69 years, 24.6% had the EGFR 19Del mutation, while 75.4% had the EGFR 21L858R mutation. Further analysis revealed that for patients aged ≤69 years without brain metastases (node 3), 37% presented with the EGFR 19Del mutation and 63% with the EGFR 21L858R mutation. Conversely, in patients with brain metastases (node 4), 45.1% had the 19Del mutation, and 54.9% had the 21L858R mutation.

### 3.4 Comparison of the predictive performance and diagnostic efficacy of the two models

**Figure 5:**
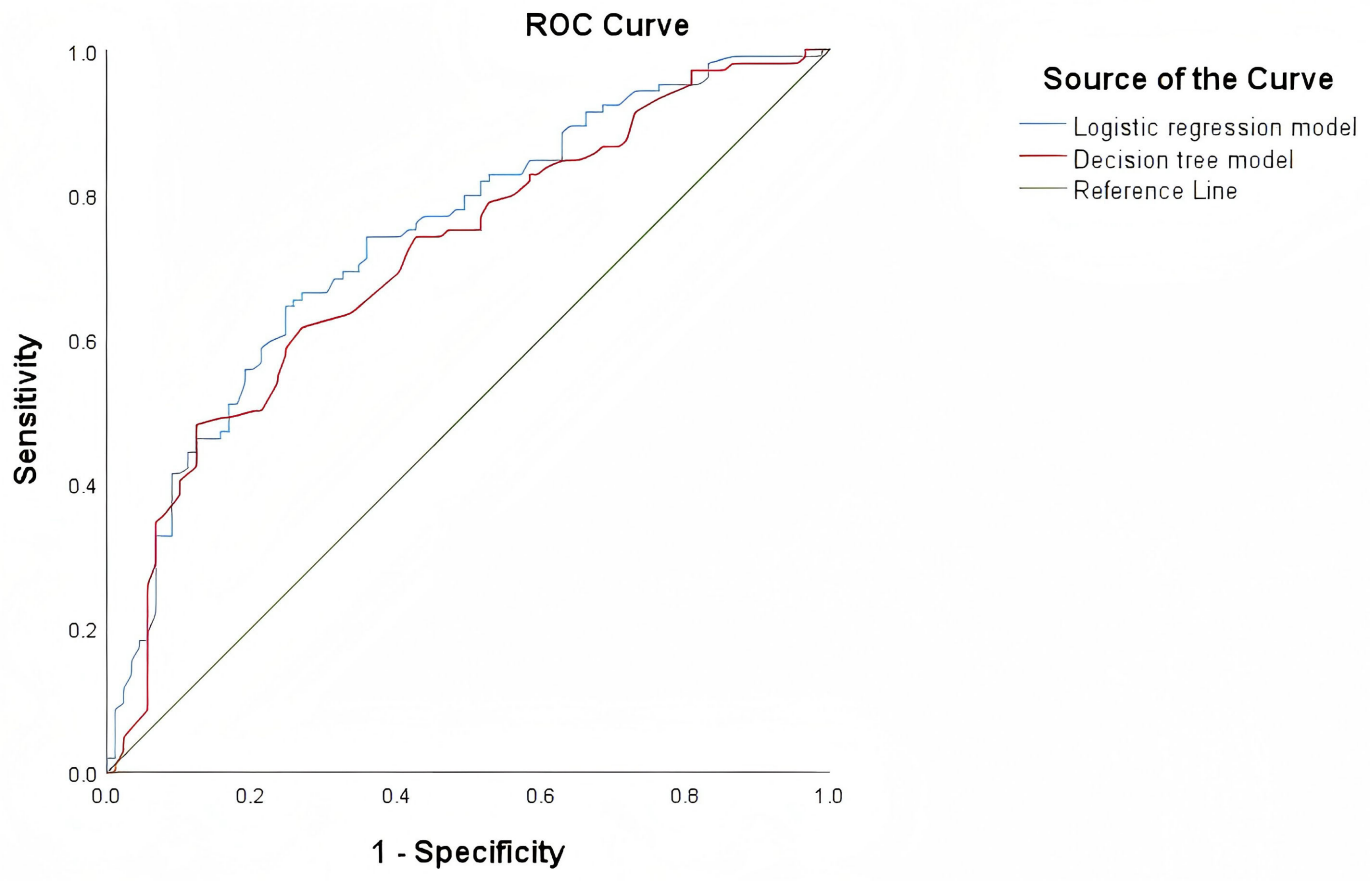
ROC curves of the decision tree model and logistic regression model for identifying 19DeL and 21L858R mutations.

The area under the curve (AUC) of the decision tree model and logistic regression model for identifying EGFR 19Del and 21L858R mutations were 0.712 (95% CI = 0.639-0.785) and 0.740 (95% CI = 0.671-0.810), respectively. The ROC curves of these two models are shown in **Figure 5**. The NRI and IDI values for diagnostic efficacy evaluation are 0.498 (*P*<0.001, 95% CI: 0.238-0.758) and 0.043 (*P* = 0.004,95% CI: 0.013-0.072), respectively. The P values for both are<0.05, indicating a significant improvement in the diagnostic ability of the logistic regression model compared to the decision tree model. However, DeLong’s test results showed no statistically significant difference between the two models (Z = 1.314, *P* value=0.189), as shown in **Table 3**.

**Table 3:** Comparison of prediction performance of DeLong test decision tree model and logistic regression model.

Test result variable(s)	AUC (95%CI)	*P* Value	NRI (95%CI)	*P* Value for NRI	IDI (95%CI)	*P* Value for IDI
Decision tree mode	0.712 (0.639-0.785)	<0.001	Reference	Reference	Reference	Reference
Logistic regression model	0.740 (0.671-0.810)	<0.001	0.498 (0.238-0.758)	0.001	0.043 (0.013-0.072)	0.004
Delong test	0.028 (0.070-0.014)	0.189	*NA*	*NA*	*NA*	*NA*

## 4 Discussion

With the continued advancement of molecular biology, the treatment paradigm for lung adenocarcinoma has shifted from conventional approaches based on histopathological and clinical characteristics toward precision medicine guided by individual genetic alterations—most notably, mutations in the epidermal growth factor receptor (EGFR) gene. Among EGFR mutations, exon 19 deletions (19Del) and the exon 21 L858R point mutation (21L858R) represent the two most prevalent subtypes, together accounting for the vast majority of EGFR-mutant lung adenocarcinomas (18, 19). Both subtypes have demonstrated substantial sensitivity to EGFR tyrosine kinase inhibitors (TKIs); however, emerging evidence suggests notable differences in their molecular profiles, drug resistance mechanisms, clinical behavior, and therapeutic responses (20, 21).Despite the frequent clinical practice of treating 19Del- and 21L858R-mutant lung adenocarcinomas with similar TKI-based regimens, studies have increasingly shown that patients harboring 19Del mutations tend to experience longer progression-free and overall survival compared to those with EGFR 21L858R mutation, particularly in the setting of advanced disease (22–24). In light of these findings, the present study aimed to develop logistic regression and decision tree models leveraging clinical and radiological features to distinguish between 19Del and 21L858R subtypes. This approach seeks to enhance individualized therapeutic planning, especially in patients with indeterminate or unavailable EGFR genotyping.

Both modeling approaches identified age and presence of brain metastasis as key predictors of EGFR mutation subtype. Consistent with prior literature, our results indicate that patients with EGFR 19Del mutation tend to be younger than those with EGFR 21L858R mutation (25). The EGFR 19Del mutation rate declined with increasing age, whereas EGFR 21L858R mutations were infrequent in patients aged ≤40 years, highlighting a clear difference in age-related mutation distribution. In our cohort, the median age of patients with EGFR 19Del and 21L858R mutations was 58.0 (IQR: 51.0–67.0) and 69.0 years (IQR: 58.0–73.0), respectively (z = 4.437, P < 0.01), corroborating previously reported trends. Brain metastasis also emerged as a distinguishing clinical feature. Among patients with EGFR 19Del mutation, 28 of 89 (31.5%) had brain metastases, compared to 51 of 104 (49.0%) in the 21L858R group—a statistically significant difference (χ² = 6.129, P = 0.013). This aligns with prior studies suggesting a higher propensity for brain dissemination in tumors harboring EGFR 21L858R mutation (26, 27). The underlying mechanism may involve increased tumor aggressiveness, enhanced permeability of the blood-brain barrier, and immune evasion capabilities associated with EGFR 21L858R-driven malignancies.

In addition, the logistic regression model identified pleural thickening and lymphadenopathy as significant predictors. Our findings concur with those of Zhang et al. (28), who reported a positive association between lymphadenopathy and the EGFR 19Del mutation. However, while Kong et al. (29) observed a stronger link between pleural thickening and EGFR 19Del mutation, our results suggest a contrary trend, warranting further investigation. Notably, these features were not selected as key decision points in the decision tree model—potentially due to differences in model architecture. Logistic regression, a parametric linear model (30), excels in quantifying direct associations between predictors and outcomes, even with relatively small or high-dimensional datasets. In contrast, decision trees operate via hierarchical segmentation and may prioritize variables with stronger or earlier splitting power, potentially overlooking subtler associations (31).

Despite these methodological differences, both models demonstrated utility in predicting EGFR mutation subtype. The logistic regression model identified four explanatory variables, whereas the decision tree model selected two. Both consistently highlighted age and brain metastasis as the most influential predictors. In terms of performance, the logistic regression model yielded an AUC of 0.740 (95% CI: 0.671–0.810), slightly outperforming the decision tree model (AUC = 0.712; 95% CI: 0.639–0.785). Net reclassification improvement (NRI) and integrated discrimination improvement (IDI) analyses further supported the superior classification performance of the logistic regression model (NRI = 0.498, P = 0.001; IDI = 0.043, P = 0.004).

However, DeLong’s test showed no statistically significant difference between AUCs (Z = 1.314, P = 0.189), suggesting that while logistic regression may offer nuanced advantages in certain contexts, both models are comparably effective overall. The discrepancy in statistical significance across performance metrics reflects their differing emphases: while NRI and IDI capture shifts in classification performance, particularly for individuals near decision thresholds, the DeLong test evaluates overall discriminative capacity. Therefore, integrating both modeling strategies may offer complementary strengths, enhancing predictive accuracy and clinical utility.

Notwithstanding these promising findings, several limitations warrant acknowledgment. First, this retrospective analysis focused exclusively on CT imaging and select clinical parameters, potentially omitting other relevant variables. Second, despite employing a double-blind approach to radiological interpretation, selection bias may have influenced the observed associations. Most importantly, while robust internal validation was performed through cross-validation techniques (applied to the decision tree model), the lack of external validation represents a key limitation that may affect the generalizability of our results. This constraint, common in radiomics studies, highlights the critical need for future prospective multicenter validation to confirm the clinical applicability of our models.

In summary, logistic regression and decision tree models constructed using age, lymphadenopathy, pleural thickening, and brain metastasis show promise for noninvasive prediction of EGFR 19Del and 21L858R mutation subtypes in lung adenocarcinoma. These models may inform genotype-tailored treatment decisions, facilitate early identification of high-risk patients, and ultimately improve prognostic assessment in clinical practice.

## Data Availability

The original contributions presented in the study are included in the article/**Supplementary Material**. Further inquiries can be directed to the corresponding author/s.
