# Supplementary material for: Noninvasive prediction of EGFR 19Del and 21L858R subtypes in lung adenocarcinoma: a comparative study of logistic regression and decision tree models
Source: Front Oncol. 2025 Sep 18;15:1642253. doi: 10.3389/fonc.2025.1642253 (PMC12488440; doi:10.3389/fonc.2025.1642253)
Supplement: Supplementary file 1 [file Table1.docx]

Table S1 **Interobserver agreement assessment of clinical and qualitative CT imaging features using Cohen's Kappa statistic**

| Variable | Kappa value |
| --- | --- |
| Gender | 1.00 |
| Age | 1.00 |
| Smoking history | 1.00 |
| Tumor location | 0.854 |
| Tumor size | 0.927 |
| Lobular sign | 0.740 |
| Spike sign | 0.893 |
| Edge | 0.793 |
| Air bronchogram | 0.841 |
| Bubble sign | 0.825 |
| Peripheralemphysema | 0.875 |
| Vascular clustering | 0.715 |
| Necrosis in tumor | 0.906 |
| Pleural traction | 0.741 |
| Pleural thickening | 0.812 |
| Calcificationintumor | 0.949 |
| Pleural effusion | 0.768 |
| Inanition | 0.864 |
| Lymphadenopathy | 0.819 |
| CT value | 0.932 |
| Bone metastasis | 0.942 |
| Brain metastases | 0.957 |
| Liver metastasis | 0.928 |
| Lung metastasis | 0.901 |
